# Bruton’s tyrosine kinase is at the crossroads of metabolic adaptation in primary malignant human lymphocytes

**DOI:** 10.1038/s41598-019-47305-2

**Published:** 2019-07-30

**Authors:** Bahram Sharif-Askari, Daniel Doyon, Miltiadis Paliouras, Raquel Aloyz

**Affiliations:** 1 Lady Davis Institute for Medical Research & Cancer Segal Center, Jewish General Hospital, Quebec, Canada; 2 Department of Medicine, Division of Experimental Medicine, McGill University, Quebec, Canada; 3 Department of Oncology, Faculty of Medicine, McGill University, Quebec, Canada

**Keywords:** Cancer metabolism, Preclinical research, Cancer metabolism, Cancer metabolism, Preclinical research

## Abstract

In this work we explored metabolic aspects of human primary leukemic lymphocytes that hold a potential impact on the treatment of Bruton tyrosine kinase (BTK)-driven diseases. Our results suggest that there is crosstalk between Bruton tyrosine kinase (BTK) signaling and bioenergetic stress responses. In primary chronic lymphocytic leukemia (CLL) lymphocytes, pharmacological interference with mitochondrial ATP synthesis or glucose metabolism affects BTK activity. Conversely, an inhibitor of BTK used clinically (ibrutinib) induces bioenergetic stress responses that in turn affect ibrutinib resistance. Although the detailed molecular mechanisms are still to be defined, our work shows for the first time that in primary B cells, metabolic stressors enhance BTK signaling and suggest that metabolic rewiring to hyperglycemia affects ibrutinib resistance in TP53 deficient chronic lymphocytic leukemia (CLL) lymphocytes.

## Introduction

Chronic lymphocytic leukemia (CLL), the most common leukemia in adults, is a clonal disease characterized by the accumulation of malignant B cells in the blood and lymphoid organs. Recent proteomic and metabolomic studies suggest that interference of glutamine, glucose or fatty acid metabolism hold the potential to affect therapeutic responses in CLL^1–3^. Furthermore, our team discovered that CLL cells containing the poor prognostic factors del17p or del11q (TP53 and ATM deletions, respectively) are metabolically different compared to CLL cells without these deletions^4,5^. For instance, del11q positive CLL cells were sensitive to glutaminase inhibitors, suggesting that therapies targeting glutamine metabolism could be effective in these cases. Although several aspects of metabolic rewiring in CLL lymphocytes have been explored recently (reviewed elsewhere^6–8^), metabolic plasticity regulation in these malignant lymphocytes is not well understood.

BTK is a TEC kinase family member driving oncogenic signaling in several B-cell malignancies including CLL^9,10^. BTK integrates intrinsic and extrinsic clues affecting survival, proliferation, adhesion and migration of B-cells^3,11–16^. We hypothesize that BTK contributes to metabolic homeostasis, defined as a balanced uptake of nutrients for macromolecule synthesis, redox control and energy production. To elucidate this relationship, our team described how ibrutinib affects glutamine and glucose uptake, depletes glutathione content and enhances ROS levels in primary CLL lymphocytes *in vitro*^11^. Recently, the inhibition of free fatty acid synthesis induced by ibrutinib was reported^14^. Notably, ibrutinib-resistant CLL lymphocytes were re-sensitized to ibrutinib by the combination of a fatty acid metabolism inhibitor and ibrutinib *in vitro*, confirming the connection of BTK signaling and fatty acid metabolism regulation^3^.

## Results and Discussion

Achievable concentrations of ibrutinib *in vivo* (≤1 µM) are cytotoxic to primary CLL lymphocytes *in vitro* in conditions enhancing BTK activity such as B-cell receptor activation or stromal cell co-culture^17^. In the absence of extrinsic BTK activation ibrutinib IC_50_ *in vitro* ranges from 0.4 to 9.7µM^18^. Accordingly, the percentage of death by 10 µM ibrutinib with respect to vehicle treated CLL lymphocytes ranges from 6.5% to 94.5%^10^. Therefore, we consider that CLL clones are ibrutinib resistant if 10 µM ibrutinib does not reduce survival by more than fifteen percent after forty eight hours with respect to vehicle-treated paired samples (i.e. Control).

**Figure 1:**
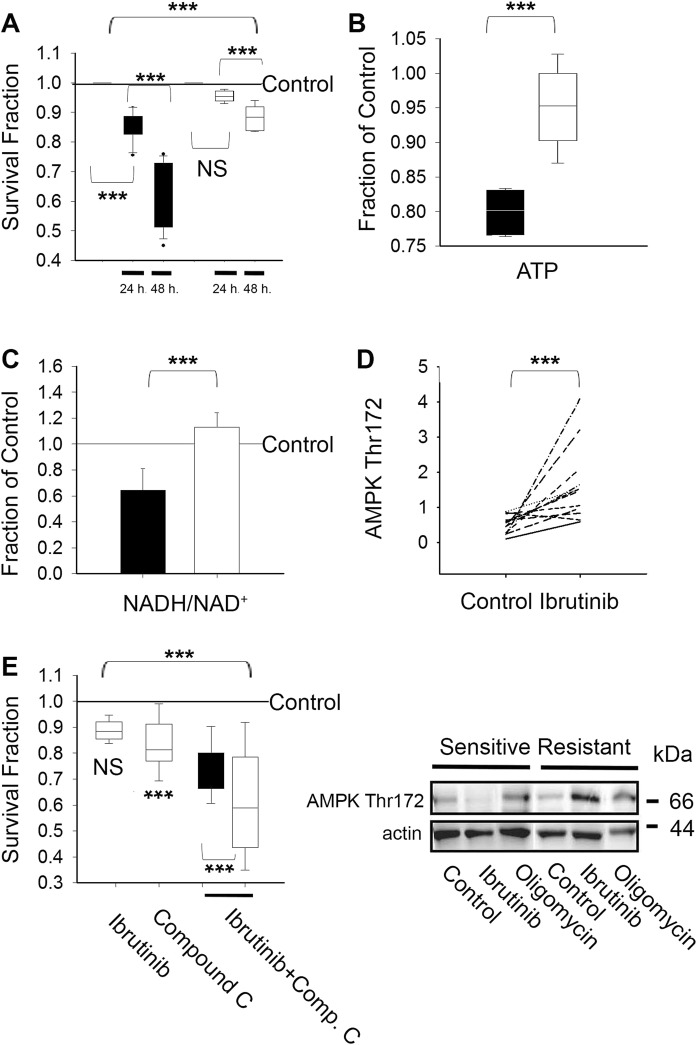
(**A**) Primary CLL lymphocyte clones sensitive (■, n = 14) or resistant to ibrutinib (□, n = 12) were treated with 10 µM ibrutinib for twenty four to forty eight hours as indicated and the values expressed as a fraction of vehicle treated paired samples (Control = 1) as described in materials and methods. The box plot graphs represent the percentiles and the median of column data. The ends of the boxes define the 25th and 75th percentiles, with a line at the median and error bars defining the 10th and 90th percentiles. Differences in the survival fractions were tested by one-way analysis of variance (ANOVA) with repeats (with Bonferroni t-test correction). NS indicates non statistical differences (p > 0.05) and ***p < 0.001. (**B**) Changes in ATP content with respect to paired vehicle treated samples were estimated in primary CLL lymphocyte clones sensitive (■, n = 4) and resistant to ibrutinib (□, n = 4) after being treated with 10 µM ibrutinib for twenty four hours. The box plot graphs represent the percentiles and the median of column data. The ends of the boxes define the 25th and 75th percentiles, with a line at the median and error bars defining the 10th and 90th percentiles. The median values were compared by two-tailed indexed t-test (95 percent two-tailed confidence interval for difference of means: −0.233 to −0.0268) with ***p = 0.022. (**C**) NADH/NAD levels with respect to paired vehicle-treated samples were obtained after treatment with 10 µM ibrutinib for twenty four hours in ibrutinib sensitive (■, n = 7) and ibrutinib resistant (□, n = 6) CLL clones. The results were obtained as described in materials and methods, normalized to viable lymphocyte numbers and expressed as the mean value ± SD. The survival fractions were normalized to vehicle treated paired samples (reference line, Control). Differences in the NADH/NAD^+^ ratios after ibrutinib treatment were assessed by two-tailed indexed t-test (95 percent two-tailed confidence interval for difference of means: −0.969 to −0.00791) and ***p = 0.047. (**D**) AMPK Thr172 was assessed twenty fours hours after vehicle (Control) or 10 µM ibrutinib treatment in ibrutinib resistant clones (n = 11) using western blot analysis as described in materials and methods. Differences in AMPK Thr 172 were assessed by paired t-test (95 percent two-tailed confidence interval for difference of means: −1.870 to −0.390) with ***p = 0.0064. Representative results are shown below the graph. Equal protein loading was confirmed by reprobing with an α-actin antibody. (**E**) The observed (□) and expected (■) effect of compound C in the survival of ibrutinib treated CLL clones were assessed twenty four hours after treatment as described in materials and methods. CLL clones resistant to ibrutinib (n = 6) were treated with 10 µM ibrutinib and 5 µM compound C alone or in combination as indicated. The box plot graphs represent the percentiles and the median of column data. The ends of the boxes define the 25th and 75th percentiles, with a line at the median and error bars defining the 10th and 90th percentiles. The survival fractions were normalized to survival in vehicle-treated paired samples (reference line, Control = 1). Comparison in the survival between groups by one-way ANOVA with repeats (Bonferroni) was significant with a ***p < 0.001. Compound C alone vs. Control, ***p = 0.002 and Ibrutinib vs. Control, ^NS^ p > 0.05. Compound C plus ibrutinib, observed (□) vs. expected (■), ***p = 0.048.

In the experiments we discuss below, CLL clones were treated with ibrutinib for no longer than twenty four hours in order to reduce biases due to mitochondrial dysfunction caused by cell death induction (Fig. 1A). Of note, 10 µM ibrutinib has been reported to inhibit not only BTK but also other TEC family members^19^. However, BTK is the major TEC kinase expressed in CLL lymphocytes and therefore BTK is a bona fide target of ibrutinib in these cells. Notably, acquired resistance to ibrutinib in CLL is due to mutations in BTK or its downstream target PLCγ^20,21^. Importantly, we focus our efforts on the investigation of a possible relationship between intrinsic BTK signaling and metabolic stress responses. Therefore we utilized CLL lymphocytes donated by ibrutinib naïve CLL patients maintained in conditions that do not enhance BTK activity.

Because recent reports indicate that ibrutinib affects metabolite utilization in CLL lymphocytes, possibly at the mitochondrial level^3,11,14^, we monitored the effect of 10 µM ibrutinib on the redox state of nicotinamide adenine dinucleotide (NAD) and on ATP content in viable CLL lymphocytes after treatment. Twenty four hours after ibrutinib treatment, ATP content in ibrutinib sensitive CLL clones was reduced with respect to ibrutinib resistant clones (Fig. 1B). The difference in ATP content between subsets after treatment, although modest, was accompanied by a forty percent decrease in ibrutinib sensitive CLL clones of reduced NAD (i.e. lowered NADH/NAD^+^ ratio) (Fig. 1C). These results suggest that reduced ATP content in ibrutinib sensitive clones is perhaps associated with reduced NAD^+^ regeneration at the TCA cycle level. Reductions in the ATP/AMP ratio, often due to decreased ATP levels, can result in bioenergetic imbalances leading to AMPK activation by phosphorylation at Thr172^22^. In order to restore bioenergetic homeostasis, activated AMPK promotes catabolism and limits anabolism enhancing ATP synthesis while reducing ATP consumption^23^. We therefore compared AMPK activity in CLL lymphocytes treated with vehicle (Control) or 10 µM ibrutinib by monitoring changes in AMPK Thr172. Twenty four hours after treatment, AMPK Thr172 was significantly heightened in ibrutinib resistant clones but not in sensitive clones (Fig. 1D and supplementary data). These results indicate that ibrutinib induces bioenergetic stress responses in primary CLL lymphocytes and suggest that efficient metabolic rewiring to ibrutinib requires AMPK dependent metabolic re-programming. Of note, ibrutinib and Oligomycin, an inhibitor of mitochondrial ATP synthesis, increased AMPK Thr172 to a similar extent (Fig. 1D lower panel and supplementary data). In addition, in agreement with previous reports demonstrating that BTK is the major TEC kinase expressed in CLL lymphocytes, our results suggest that ibrutinib induces metabolic stress when utilized not only at 10 µM but also at lower concentrations reported to inhibit BTK^19^. Treatment with 0 to 1 µM ibrutinib for twenty four hours enhances AMPK Thr172, including a clinically achievable concentration of ibrutinib (200 nM) and the Cmax of the drug (1 µM)^17^ (supplementary data).

We reasoned that curtailing AMPK activity might sensitize ibrutinib resistant clones to ibrutinib. To test the role of AMPK activation on ibrutinib resistance, we utilized the AMPK inhibitor, compound C. While 10 µM ibrutinib was not cytotoxic to ibrutinib resistant CLL clones, 5 µM compound C reduced survival by twenty percent. Reduced survival by treatment with compound C alone of unstimulated peripheral CLL cells *in vitro* is in line with the reported role of AMPK in metabolic rewiring to quiescence in other cell types^23,24^. Additionally, the survival fraction after ibrutinib plus compound C treatment (i.e. observed survival fraction) was significantly lower compared with the expected behaviour of the combination by the effect of each individual inhibitor^25,26^ (Fig. 1E), suggesting that resistance to ibrutinib involves AMPK-mediated energetic re-programming. In agreement with this scenario, we previously reported that inhibition of mitochondrial fatty acid oxidation (a process favored by AMPK signaling) reduces ibrutinib resistance in CLL lymphocytes^3^. Notably, we find that resistance to a multi tyrosine kinase inhibitor hindering BTK upstream signaling^27^ is associated as well with AMPK bioenergetic re-programming. Specifically, we reported that either compound C or an inhibitor of fatty acid oxidation reduces dasatinib resistance in CLL clones refractory to dasatinib^12^.

**Figure 2:**
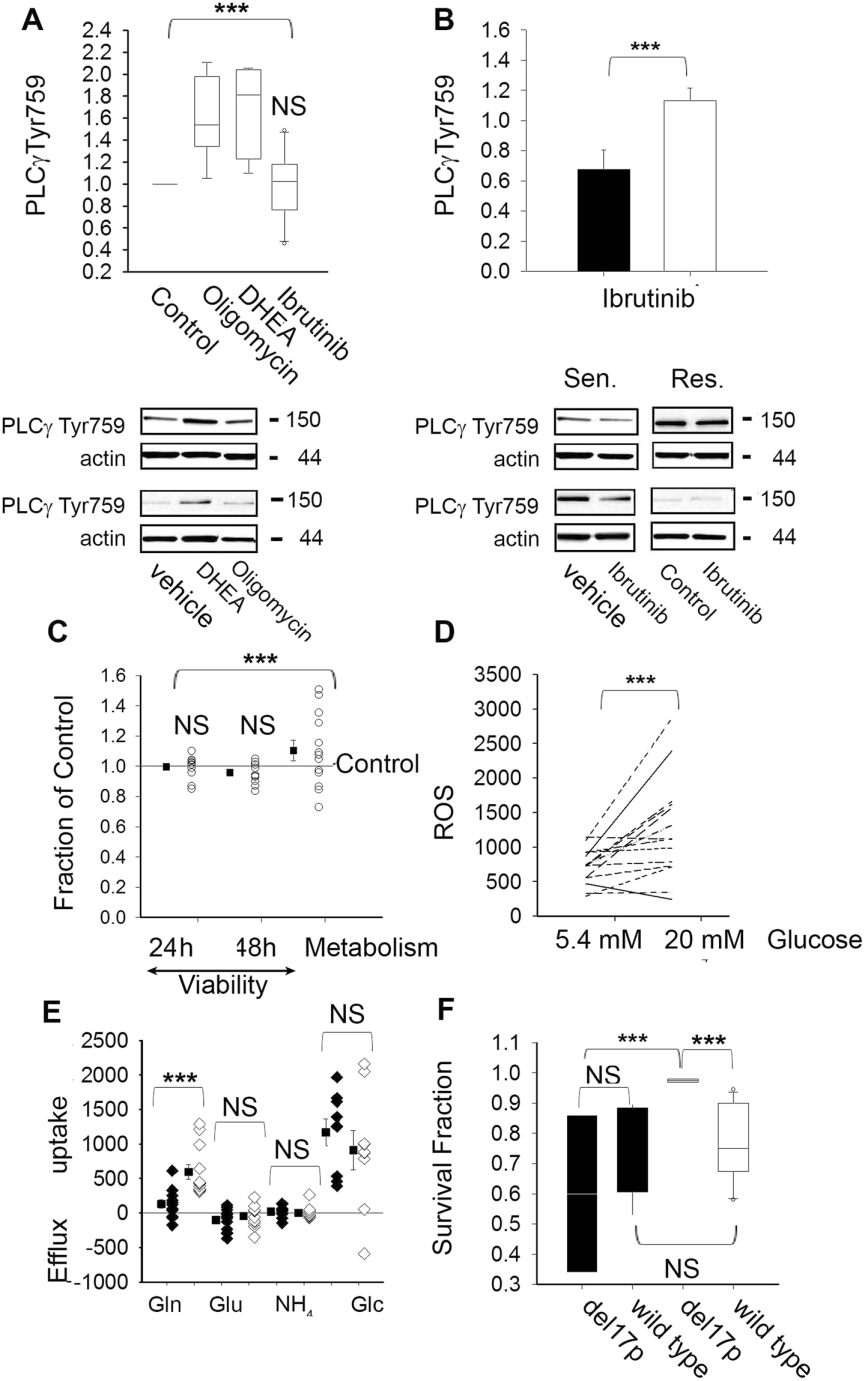
(**A**) PLCγ phosphorylation at Tyr759 was assessed by western blot as described in materials and methods in primary CLL lymphocytes sensitive (n = 5) and resistant (n = 5) to ibrutinib forty eight hours after treatment with vehicle (Control), ibrutinib (10 µM), DHEA (10 µM) or Oligomycin (2 µM). The values were normalized by actin signal and expressed as the fraction of paired vehicle treated samples. The box plot graphs represent the percentiles and the median of column data. The ends of the boxes define the 25th and 75th percentiles, with a line at the median and error bars defining the 10th and 90th percentiles. Differences in the median values with respect to Control (1) were assessed by one-way ANOVA with repeats (Bonferroni test) (Power of performed test with alpha = 0.050: 1.000) and p < 0.001. Representative results are shown below the bar graph and scans of the original western blots are provided in the supplementary figure. (**B**) PLCγ phosphorylation at Tyr759 after ibrutinib treatment and normalized by the signal in paired vehicle treated samples. The bar graph represents the mean value ± SD in ibrutinib sensitive clones (■, n = 5) and ibrutinib resistant clones (□, n = 5). The mean values were compared by two tailed t-test (t = −3.112 with 9 degrees of freedom. 95 percent two-tailed confidence interval for difference of means: −0.855 to −0.135) and ***p = 0.0125. Representative results are shown below the bar graph and scans of the original western blots are provided in the supplementary figure. (**C**) Survival and metabolic activity in 20 mM glucose and 5.4 mM glucose (Control = 1) were compared in CLL clones sensitive and resistant to ibrutinib (n = 13) by one-way ANOVA with repeats (Bonferroni test, Power of performed test with alpha = 0.050: 0.985). The graph represents the survival of individual clones (○) and mean survival values (■) ± SE. NS indicates non-significant differences (p > 0,05) and ***P < 0.001. (**D**) Basal ROS levels in viable lymphocytes were assessed by flow cytometry in sixteen CLL clones as described in materials and methods in a physiologic glucose (Glc) concentration (5.5 mM) or in hyperglycemia (20 mM Glc). Basal ROS levels in the two conditions were compared by paired t-test in primary CLL clones (n = 16) (95 percent two-tailed confidence interval for difference of means: −802.054 to −100.825) with ***p = 0.0151. (**E**) Metabolite uptake (positive numbers) and extracellular accumulation or efflux (negative number) of glutamine (Gln), glutamate (Glu), ammonia (NH4^+^) and glucose (Glc) were assessed utilizing a Nova Bioprofile Analyzer 400 and normalized by viable cell numbers in primary CLL clones (n ≥ 8). Gln, Glu, NH4^+^ uptake and efflux in physiologic glucose (♦, 5.5 mM) or hyperglycemia (◊, 20 mM), ■ represent the mean value ± SE and were compared by two-tailed paired t-test, NS represents p > 0.05. ***p** = **0.000585 for Gln (t = −4.766 with 11 degrees of freedom, 95 percent two-tailed confidence interval for difference of means: −677.348 to −249.372); ^NS^p = 0.316 for Glu t = −1.052 with 11 degrees of freedom. 95 percent two-tailed confidence interval for difference of means: −180.879 to 63.914). (**F**) Differences in survival fractions after 10 µM ibrutinib treatment in physiologic (■) or hyperglycemic (□) conditions were obtained as described in materials and methods in paired samples of CLL clones positive (n = 3) or negative (n = 11) for del17p (e.g. wild type). The box plot graphs represent the percentiles and the median of column data. The ends of the boxes define the 25th and 75th percentiles, with a line at the median and error bars defining the 10th and 90th percentiles. The survival fractions in physiologic glucose or hyperglycemia between del17p and wild type CLL clones were compared using Kruskal-Wallis one-way ANOVA on ranks. NS indicates p > 0.05 and ***p = 0.048.

We next explored the existence of feedforward loops between BTK and metabolic stress responses in primary CLL lymphocytes. If so, conditions affecting metabolic homeostasis should affect BTK signaling. We reasoned that if metabolic stress responses affect BTK signaling, non-cytotoxic concentrations of metabolic stressors have the potential to affect the phosphorylation of BTK downstream targets. To test this hypothesis, we monitored for changes in the phosphorylation of PLCγ Tyr759^28,29^ in primary CLL lymphocytes challenged with non-cytotoxic concentrations of compounds affecting metabolic fluxes or bioenergetics in primary CLL lymphocytes^6–8^. Twenty four hours after treatment with an inhibitor of mitochondrial ATP synthesis (2.5 µM oligomycin) or an inhibitor of glucose utilization via the pentose phosphate pathway (10 µM DHEA), PLCγ Tyr759 increased significantly with respect to vehicle treated paired samples regardless of ibrutinib cytotoxicity (Fig. 2A upper panel). Representative results are shown in the lower panel of Fig. 2A and in the supplementary material. Notably, twenty four hours after treatment, ibrutinib did not significantly affect PLCγ Tyr759 with respect to vehicle treated paired samples (Control). However, when the results were segregated vis a vis ibrutinib resistance, PLCγ Tyr759 was significantly lower in ibrutinib sensitive clones with respect to resistant clones after 10 µM ibrutinib treatment (Fig. 2B). Representative results are shown in the lower panel of Fig. 2B and in the supplementary data. In premalignant B-cells, PLCγ activity is affected by feedforward loops triggered by metabolic stress signals through Myc signaling. In this scenario, Myc overexpression has been shown to maintain B-cell receptor signaling and BTK activity despite treatment with ibrutinib, and therefore contributing to ibrutinib resistance^30^. In CLL, Myc signaling has been associated with aggressive disease and metabolic rewiring to stromal cell microenvironments^31,32^. Whether or not Myc plays a role in feedforward activation of B-cell receptor signaling by metabolic stress in CLL lymphocytes is yet to be defined.

Acknowledgement that stress can be generated by supraphysiological nutrient availability has recently led to the formulation of culture media containing nutrients in the physiological range^33^. Although it is still a controversial issue, hyperglycemia associated with type 2 diabetes has been linked with elevated incidence of certain types of cancer^34^. In proliferating cancer cell lines, supraphysiological concentrations of nutrients affects metabolic rewiring without affecting proliferation. However, nutrient availability has the potential to affect the efficacy of compounds hindering targets involved in metabolic stress responses^33,35^. Although culture media formulations often include supraphysiological concentrations of metabolites, the effect of excess nutrients in primary cancer cells and particularly in quiescent cancer cells such as peripheral CLL lymphocytes has not been explored. In light of the discussion above, it is important to mention that we have previously reported that metabolic rewiring in response to excess or limiting glucose in culture media affects dasatinib sensitivity in primary CLL cells and therefore the results we reported here were obtained utilizing a physiologic concentration of glucose (5.4 µM)^12^. In quiescent primary CLL lymphocytes neither survival nor metabolic activity was affected by hyperglycemia (i.e. 20 mM glucose) in the samples tested (Fig. 2C). In contrast, ROS levels were significantly elevated by hyperglycemia with respect to physiologic glucose (Fig. 2D). Elevated ROS production by hyperglycemia has been linked to imbalances between ROS production and detoxification processes^36^. Our results indicate that regardless of the mechanism involved, enhanced ROS by metabolic rewiring to hyperglycemia does not compromise survival of primary CLL lymphocytes *in vitro*.

As recently reported in proliferating human cancer cell lines^33^, glucose uptake was not affected by excessive glucose but resulted in heightened glutamine uptake (Fig. 2E). In addition to changes in glucose and glutamine uptake, we assessed for changes in extracellular levels of glutamate and ammonia because these metabolites are by-products of amino acid metabolism.

Extracellular glutamate and ammonia accumulation were not affected by hyperglycemia, suggesting that amino acid oxidation is not affected by excess glucose availability. However, we can’t rule out a shift on glutamate and ammonia production by other pathways in hyperglycemic conditions because these two metabolites are as well by-products of the hexosamine biosynthetic pathway, reportedly upregulated in CLL B cells^37^.

Hyperglycemia does not affect survival, BTK activity or bioenergetics homeostasis at the time point tested (24 h) because PLCγ Tyr759 nor AMPK Thr172 were affected by this condition (data not shown). Consistently, ibrutinib cytotoxicity was not affected by hyperglycemic conditions. However, when we included CLL clones with deficient TP53 signaling (del17p), ibrutinib resistance was increased with respect to normoglycemic conditions (Fig. 2F). Approximately ten percent of CLL cases display a hemizygous deletion encompassing the TP53 locus (del17p). Del17p cases show faster progression and inferior therapeutic responses to available treatments, including ibrutinib therapy^4,5,18^. The mechanism involved in enhanced resistance to ibrutinib in del17p clones is still to be defined. Metabolic traits reported in TP53 deficient cells might contribute to elevated resistance to ibrutinib by hyperglycemia in del17p CLL. For example, deficient TP53 could potentially affect glucose fluxes and mitochondrial oxidation in del17p clones following ibrutinib treatment^8^. Altered mitochondrial metabolism has been reported in del17p clones, which are characterized by heightened mitochondrial biogenesis^38^. Beyond the molecular mechanisms involved, our results suggest that non-controlled metabolic disorders might affect ibrutinib responses in patients with altered TP53 signaling.

## Materials and Methods

### Patient samples

To analyze the metabolism related to ibrutinib resistance in CLL, we collected peripheral blood samples from 30 patients with a diagnosis of B-CLL at the Jewish General Hospital. Unless otherwise indicated, del11q and del17 cases were excluded from the study. CLL lymphocytes were isolated by Ficoll-Hypaque. This study was carried out in accordance with the recommendations of the published guidelines of the TCPS2 – Tri-Council Policy Statement: Ethical Conduct for Research Involving Humans (2014), Jewish General Hospital Research Ethics Committee. The protocol was approved by the Jewish General Hospital Research Ethics Committee. All subjects gave written informed consent in accordance with the Declaration of Helsinki. All CLL patients were ibrutinib naïve.

### Cell culture, and compound treatments

Primary CLL cells, from our cell bank, were thawed and cultured overnight in AIM-V (GIBCO, 12055–091), 10% FBS at 37 °C and 5% CO_2_. CLL cells were then seeded and treated in RPMI modified media containing physiologic glucose concentration (5.5 mM), 10% FBS, 25 mM HEPES, 100 U/mL penicillin/streptomycin, at 37 °C and 5% CO_2_. To determine the influence of glutamine availability in CLL lymphocytes, they were cultured in the presence or absence of 5 mM glutamine. The compounds used to treat the cells for 24 h or 48 h were Ibrutinib (Selleckchem, S2680), Oligomycin A (Sigma, 75351), DHEA (Sigma, D-063).

### Viability and metabolic assay

To determine the presence of metabolically active viable cells, we used a Metabolic Activity Dead Cell Apoptosis Kit (Invitrogen, V35114) following manufacturer instructions. CLL cells were collected 48 h after treatment, and spun down at 1300 rpm at 4 °C for 5 min. Then cells were washed in 1X Annexin binding buffer (Invitrogen, V13246), spun down at 1300 rpm at 4 °C for 5 min, re-suspended in 250 µL of 1X Annexin binding buffer and stained with Sytox green, C12-Resazurin, and APC-Annexin V. Data was collected by flow cytometry in a BD FACSAria Fusion cell sorter at 561–582/15 nm (C12-Resazurin), 488–530/30 nm (Sytox green), 640–670/30 nm (APC-Annexin V). Data was analyzed with FlowJo V.10 software (Systat Software Inc.). To obtain the expected cytotoxicity by the combination of compound C and ibrutinib we utilized an algorithm that greatly reduces the number of experiments needed in order to reduce the use of non-renewable sources such as primary CLL lymphocytes. Using this model the expected cytotoxicity of a drug combination is obtained as the product of the cytotoxicity obtained with each individual compound^25,26^.

### Extracellular glucose, glutamine, glutamate, and ammonia quantification

After 24 h treatment, cell media was collected for metabolite quantification in a NOVA Bioprofile Analyzer 400 at GCRC Metabolomics Core (McGill University). Glucose, glutamine, glutamate, and ammonia uptake (or secretion) were estimated by subtracting their concentrations in conditioned media from their concentrations in fresh media and normalized to viable cell number (µM/10^6^ cells). In addition, Nova Bioprofile 400 documented the pH of conditioned media for each sample. The pH was not affected by any treatment in any of the tested samples.

### ROS level determination

After 24 h treatment, a CellROX® Green Flow Cytometry Assay Kit (Invitrogen, C10492) was used as directed by the manufacturer. Data was collected by flow cytometry in a BD FACSAria Fusion cell sorter at 488–530/30 nm (CellRox) and 640–670/30 nm (Sytox red). The cell viability at 24 h treatment was quantified using Sytox red. Data was analyzed with FlowJo V.10 software (Systat Software Inc.).

### NAD/NADH determination

Oxidized and reduced NAD ratio was determined with NAD/NADH-Glo Assay (Promega, G9071 [NADH/NAD^+^]). 1.5 × 10^6^ viable cells were treated with selected compounds for 24 h. After treatment, cells were collected, re-suspended in 40 µL of 1X PBS, and treated to determine NAD (H) individually according to manufacturer instructions. Measurements were obtained after 1 h incubation of the reaction, using a FluoStar Optima Reader. The NAD/NADH ratio was determined using the luminescence signal obtained, since it is proportional to the NAD amount in the sample.

### Immunoblotting analysis

Protein samples were prepared by collecting cell pellets 24 h after treatment. Protein total cell lysates were prepared and fractionated on 4–12% Criterion™ XT Bis-Tris gels (Bio-Rad, 3450124) in 1 × -XT MOPS buffer (BioRad, 161–0788), and transferred to 0.22 µm nitrocellulose membranes (Bio-Rad, 1620112). After transfer, the membranes were cut in three strips corresponding to the expected migration of the targets of interest and probed with the corresponding specific antibodies. We determined the protein expression of PLCγ Tyr759 (CST, 3872), AMPK Thr172 (Santa Cruz, sc-33524), and β-Actin (Santa Cruz, sc-1616). Western Blot bands quantification were performed using ImageJ 1.49 v software (NIH, USA).

### Data analysis

Comparison between pairs were performed using two-tailed paired t-test or t-test analyses and multiple Comparison Procedures (Bonferroni t-test) using Sigma Plot version 13.0. All tests were performed with α and statistical power equal or greater than 0.5 and 90 percent respectively. Differences were considered significant if the p value was < 0.05.

## Supplementary information

Suplementary Data
