# Supplementary material for: Bruton’s tyrosine kinase is at the crossroads of metabolic adaptation in primary malignant human lymphocytes
Source: Sci Rep. 2019 Jul 30;9:11069. doi: 10.1038/s41598-019-47305-2 (PMC6667467; doi:10.1038/s41598-019-47305-2)

Bruton's tyrosine kinase is at the crossroads of metabolic adaptation in primary malignant human lymphocytes

Bahram Sharif-Askari^1^, Daniel Doyon^1^, Miltiadis Paliouras^1,3^, Raquel Aloyz^1, 2,3*^

^1^Lady Davis Institute for Medical Research & Cancer Segal Center, Jewish General Hospital

^2^Experimental Medicine, Faculty of Medicine, McGill University

^3^Department of Oncology, Faculty of Medicine, McGill University

*Corresponding author: Raquel Aloyz, [raquel.aloyz@mcgill.ca](mailto:raquel.aloyz@mcgill.ca)

3755 Cote Sainte Catherine Road, Room E-439

H3T 1E2, Montreal, Quebec, Canada


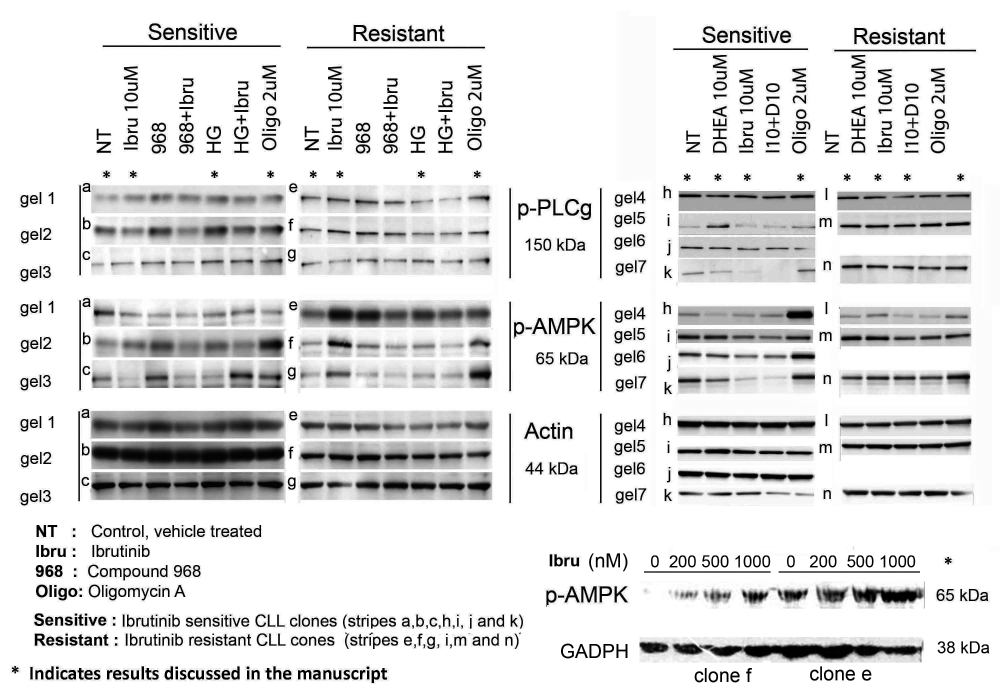


**OD plots for the corresponding western blot scans above captured with image J software**


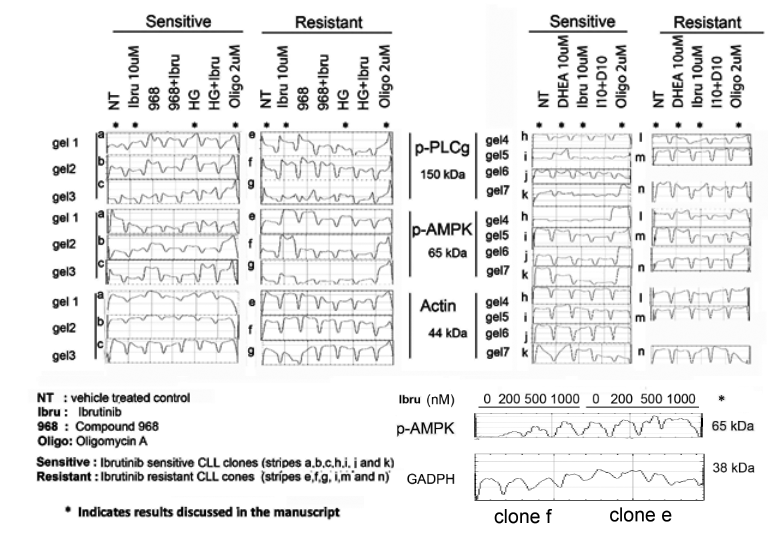

Supplement: Supplementary file 1 — Suplementary Data [file 41598_2019_47305_MOESM1_ESM.docx]
